# ILC2-mediated immune crosstalk in chronic (vascular) inflammation

**DOI:** 10.3389/fimmu.2023.1326440

**Published:** 2023-12-20

**Authors:** Maria Kral, Emiel P.C. van der Vorst, Alexey Surnov, Christian Weber, Yvonne Döring

**Affiliations:** ^1^Institute for Cardiovascular Prevention (IPEK), Ludwig-Maximilians University Munich, Munich, Germany; ^2^DZHK (German Center for Cardiovascular Research), Partner Site Munich Heart Alliance, Munich, Germany; ^3^Aachen-Maastricht Institute for CardioRenal Disease (AMICARE), Interdisciplinary Center for Clinical Research (IZKF), Institute for Molecular Cardiovascular Research (IMCAR), RWTH Aachen University, Aachen, Germany; ^4^Type 1 Diabetes Immunology (TDI), Helmholtz Diabetes Center (HDC), Helmholtz Center Munich, Munich, Germany; ^5^Department of Biochemistry, Cardiovascular Research Institute Maastricht (CARIM), Maastricht University Medical Centre, Maastricht, Netherlands; ^6^Munich Cluster for Systems Neurology (SyNergy), Munich, Germany; ^7^Department of Angiology, Swiss Cardiovascular Center, Inselspital, Bern University Hospital, University of Bern, Bern, Switzerland; ^8^Department for BioMedical Research (DBMR) Bern University Hospital, University of Bern, Bern, Switzerland

**Keywords:** ILC2, atherosclerosis, chemokine receptors, inflammation, cardiovascular disease, tissue repair

## Abstract

Crosstalk between innate and adaptive immunity is pivotal for an efficient immune response and to maintain immune homeostasis under steady state conditions. As part of the innate immune system, type 2 innate lymphoid cells (ILC2s) have emerged as new important regulators of tissue homeostasis and repair by fine-tuning innate-adaptive immune cell crosstalk. ILC2s mediate either pro- or anti-inflammatory immune responses in a context dependent manner. Inflammation has proven to be a key driver of atherosclerosis, resembling the key underlying pathophysiology of cardiovascular disease (CVD). Notably, numerous studies point towards an atheroprotective role of ILC2s e.g., by mediating secretion of type-II cytokines (IL-5, IL-13, IL-9). Boosting these protective responses may be suitable for promising future therapy, although these protective cues are currently incompletely understood. Additionally, little is known about the mechanisms by which chemokine/chemokine receptor signaling shapes ILC2 functions in vascular inflammation and atherosclerosis. Hence, this review will focus on the latest findings regarding the protective and chemokine/chemokine receptor guided interplay between ILC2s and other immune cells like T and B cells, dendritic cells and macrophages in atherosclerosis. Further, we will elaborate on potential therapeutic implications which result or could be distilled from the dialogue of ILC2s with cells of the immune system in cardiovascular diseases.

## Introduction

The first line of defense in our immune system, known as innate immunity, promptly identifies invading pathogens through the immediate recognition of pathogen-associated molecular patterns (PAMPs) present on their surfaces (1) or damage-associated molecular patterns (DAMPs) released upon tissue injury or inflammation (2). Receptor activation upon PAMP or DAMP binding leads to the initiation of signaling cascades that result in the release of pro-inflammatory cytokines and chemokines produced by cells of the innate immune system. Effective communication between innate and adaptive immune cells is a fundamental requirement for a robust immune response. This communication is partially ensured by the innate immune system instructing the adaptive immune system through the presentation of pathogen-derived peptides to adaptive immune cells or via the signaling axis involving chemokines and their receptors. Chemokines serve as chemotactic cytokines and play a crucial role in recruiting leukocytes to sites of inflammation or injury (3).

Immune cell subsets are distributed among different sites in our body where they reside in specific tissue areas or circulate via the blood stream. Thus, cell-cell interactions across different locations add another layer of complexity to this cross-talk. Most of our knowledge about the human immune system is derived from studies from human peripheral blood. However, recent advances in computational modeling as well as better access to human tissue samples has enabled us to study immune reactions and functions of different immune cells across various locations in the body in addition to peripheral blood (4–9). Recent cell populations gaining attention are for example natural killer (NK) cells or innate lymphoid cells (ILCs), both of which mainly reside in tissues [e.g. in mucosal linings or adipose tissue (AT)] and adapt to changes in their environment. ILCs are an important source of cytokines for the initiation of immune reactions and have newly been described as an important bridge between the innate and adaptive immune system (10). ILCs have been discovered as the “innate immune cell pendant” to CD4^+^ T cells, since they develop from the common lymphoid progenitor and they express signature cytokines similar to CD4^+^ T cells. ILCs comprise of three subgroups: ILC1, ILC2, and ILC3 and their main common characteristics are the lack of antigen-specific receptors and lack of markers that are expressed on immune cells of the common hematopoietic cell lineages (11).

ILCs especially reside at mucosal barriers, where they are rapidly activated in response to invading pathogens. ILC2s secrete cytokines typical for T helper cell type 2 (Th2) cells (interleukin (IL)-5, IL-4, IL-9, and IL-13) in response to IL-33, IL-25 and thymic stromal lymphopoietin (TSLP) and require expression of GATA binding protein 3 (GATA3). Importantly, ILC2s have been first discovered in AT and their critical role in regulating AT homeostasis by the release of type-2 cytokines has henceforth been intensively studied (12). In addition, ILC2s have been implicated in the development of several diseases, where they exert either pro- or anti-inflammatory properties in a tissue-dependent manner depending on the expression of specific markers. Moreover, it has been demonstrated by Huang et al. that tissue-resident natural ILC2s (nILC2) phenotypically differ from ILC2s that are activated upon infection, termed inflammatory ILC2s (iILC2S) (13). For example, they have been described to be involved in the pathophysiology of cardiovascular diseases (CVDs). CVDs consist of conditions affecting the heart or blood vessels including coronary heart disease and cerebrovascular disease. The dominant cause for this condition is atherosclerosis, the thickening of the arteries due to accumulation of lipids and immune cells resulting in plaque development. However, the exact mechanisms by which ILC2s mediate their pleitropic roles in different diseases including CVDs are not well understood.

Hence, this review will concentrate on elucidating the mechanisms through which ILC2s facilitate their multifaceted communication with various immune cells to orchestrate immune responses. We will describe their roles at different anatomical sites in the body in steady state and in diseases like asthma or skin fibrosis. Additionally, we will underscore their significant contributions to CVD and will shed light on the specific chemokine-chemokine receptor signaling pathways that play a pivotal role in mediating immune responses of ILC2s in CVD. As an outlook we will discuss how these cell-to-cell interactions during immune responses can serve as promising novel targets for therapeutic interventions in the management of chronic inflammatory disorders.

## ILC2 in tissue maintenance and repair

ILC2s express different surface markers depending on the tissue they inhabit which facilitates their tissue-specific identification. These cells are most prominently found in the skin, intestine, lung, and AT where they exert context specific functions in steady state (11, 14, 15).

Within visceral adipose tissue (VAT) for example, ILC2s primarily produce IL-5 and IL-13 in response to IL-33, leading to the recruitment of eosinophils (14, 15). These eosinophils, in conjunction with regulatory T cells (Tregs) and anti-inflammatory M2 macrophages, play crucial roles in reducing inflammation within the AT. The presence of IL-33 attracts these tissue-resident Tregs to sites of AT inflammation through ligation of its receptor interleukin 1 receptor-like 1 (IL1RL1), also known as ST2 (16). Therefore, ILC2s function as mediators for Treg recruitment via interaction with the co-stimulatory molecule OX40L expressed on ILC2s following IL-33 stimulation, a mechanism also observed in other tissues such as the lung and intestine (17). Conversely, it has been reported that during inflammatory conditions, such as obesity, the numbers of ILC2s decrease in both mouse and human adipose tissue (18, 19). This leads to a transition from anti-inflammatory M2 macrophages to pro-inflammatory M1 macrophages, marked by the release of pro-inflammatory cytokines. Consequently, studies utilizing mice deficient in ILC2s have demonstrated a significant reduction in eosinophils and M2 macrophages in VAT, ultimately leading to increased adiposity and insulin resistance in animal models (19).

Interestingly, ILC2s which are distributed along different tissues are also involved in mechanisms of tissue repair. In response to tissue damage, e.g. after cardiac stress, liver damage, or upon skin fibrosis, stromal cells release the alarmin IL-33 to recruit ILC2s via IL-33R/ST2 (20). This interaction triggers GATA3 phosphorylation, a transcription factor which is required for regulating type-2 cytokine genes (IL-4, IL-5, IL-13) (21). Moreover, the release of IL-13 by ILC2s recruits alternatively activated M2 macrophages, which have been shown to be important to initiate wound healing and tissue homeostasis (19, 22, 23).

These studies highlight that ILC2s differentially orchestrate immune responses in a tissue-specific manner under steady state and inflammatory conditions in order to maintain tissue homeostasis or to initiate tissue repair. However, depending on tissue type and disease context, ILC2s have been reported to exert dual roles of mediating either pro- or anti-inflammatory responses.

## ILC2 in inflammation and infection

Initially, ILC2s have been described to exert type-2 immunity by secreting type-2 cytokines (IL-5, IL-13) upon helminth infection (24). A sustained type-2 immunity response, however, can also lead to tissue damage and chronic inflammation. For instance, in asthma and allergic lung inflammation, ILC2s are the main producers of type-2 immune cytokines, thereby driving these pathologies. Accordingly, it has been demonstrated that ILC2-mediated IL-13-IL-33 signaling induced airway hyper-reactivity (AHR) upon influenza infection in mice (25). Conversely, blocking the IL-33/IL-33R signaling pathway or the use of anti-CD90 monoclonal antibodies to block ILC2 expansion protected mice from the influenza-induced AHR, thereby proving an essential role of ILC2s in mediating airway inflammation (25). Importantly, Halim et al. could demonstrate that ILC2s are required to initiate a Th2 cell differentiation upon intranasal papain administration, thus building a bridge from innate to adaptive immunity in allergic lung inflammation (26).

Similarly, ILC2s have also been reported to mediate pro-inflammatory immune responses in inflamed liver tissue (27). In a murine model of Con A-induced immune-mediated hepatitis, evidence could be provided for a link between increased levels of IL-33 and enhanced numbers of ILC2s, which recruited eosinophils and the sustained release of type-2 cytokines, further promoting tissue damage, as seen in the AHR mouse model. Interestingly, the study found that *a priori* IL-33 treatment before Con A challenge led to an expansion of ST2^+^ Tregs and that this specific population regulated ILC2 activity and hence, led to a reduction of liver inflammation (27).

Conversely, in a model of arthritis, ILC2s are required for the resolution of inflammation. Mechanistically, it has been shown that IL-9 triggered the activation of ILC2s which in turn recruits Tregs via interaction of the co-stimulatory molecules Glucocorticoid-induced tumor necrosis factor receptor-related protein (GITR; Tregs) - GITR ligand (GITRL; ILC2s) and Inducible T Cell Costimulator (ICOS; Tregs) - ICOS ligand (ICOSL; ILC2s) (28). Moreover, patients in remission phase of rheumatoid arthritis showed increased numbers of IL-9-expressing ILC2s in their joints and in the circulation (28).

The dual role of ILC2 subsets, i.e. promoting either pro- or anti-inflammatory responses depending on disease- and tissue-context based on the expression of specific markers as outlined earlier, is further emphasized by recent publications showing a regulatory function of ILC2s through the production of IL-10 upon exposure to the allergen papainin in the lung (29) and during intestinal inflammation (30). Treg-inducing factor, retinoic acid (RA), induced the IL-10-producing subset of ILC2s *in vitro* (31) and these regulatory ILC2s were able to mediate resolution of lung and intestinal inflammation in mouse models (29, 30). In humans, these cells were increased in human nasal tissue from healthy donors compared to patients suffering from chronic rhinosinusitis with nasal polyps (30). More recently, regulatory ILC2s have been associated with a better clinical response upon allergen-induced immunotherapy (32). In contrast to this, analysis of extracellular particles (EPs) derived from severe Coronavirus Disease of 2019 (COVID-19) patients revealed an activated phenotype of ILC2s, which led to a more aggressive disease score (33). Interestingly, EPs from severe COVID-19 patients also failed to dampen the cytokine production of ILC2s (33).

Taken together, these studies outline how environmental stimuli, the surrounding milieu, disease context and the release of specific cytokines shape ILC2 function in healthy and diseased states. Their pleiotropic function is further highlighted in low-grade inflammatory obesity (34). As outlined before, in obese patients or mice, numbers of ILC2s are reduced and thus, an inflammatory milieu prevails. However, the underlying mechanisms of an obesity-induced decrease in ILC2 numbers remain poorly understood. The pro-inflammatory environment in obesity is associated with an increased risk of developing cardiovascular disorders. Therefore, we will shed light on the consequence of ILC2 dysregulation in inflammatory-induced CVDs.

## ILC2 in atherosclerotic cardiovascular diseases

CVDs are the leading cause of death worldwide and they comprise of coronary heart diseases, peripheral artery disease, stroke, and cerebrovascular diseases, among others. Herein, atherosclerosis is the underlying pathological process which arises from the accumulation of lipids and immune cells in the intima of arteries, forming so called atherosclerotic plaques, leading to an increased thickness and stiffness of the vessels. Besides non-modifiable risk factors like increasing age, gender or genetic factors, several modifiable risk factors exist for atherosclerosis, like smoking, hypertension and hyperlipidemia. Therefore it is not surprising that obesity, characterized by excessive lipid accumulation and hyperlipidemia, is also a major risk factor for atherosclerotic cardiovascular diseases (ACVDs), although the underlying mechanisms explaining this association are still not completely understood.

Retention of lipids causes the modification of low-density-lipoprotein (LDL) into oxidized-LDL (oxLDL), which is targeted by the immune system as “non-self”. Moreover, obesity-induced chronic inflammation is caused by elevated serum levels of pro-inflammatory cytokines, which further attracts leukocytes to infiltrate the lesion (35).

**Figure 1:**
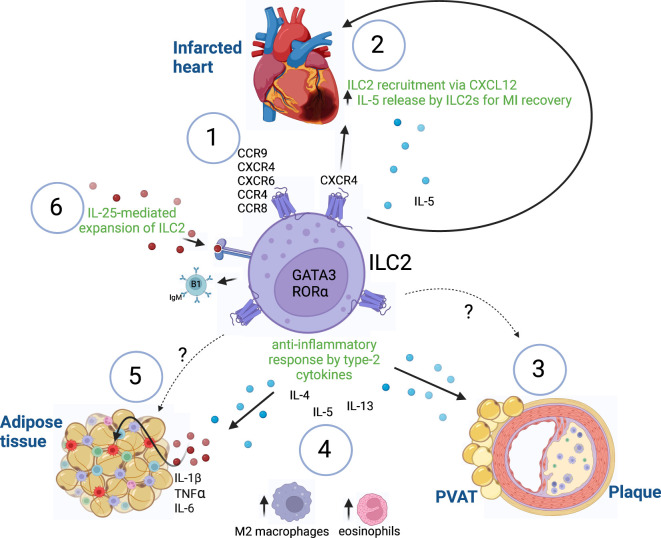
ILC2-mediated immune responses. (1) Expression of known chemokine receptors on ILC2s. (2) Recruitment of ILC2s after myocardial infarction via CXCR4/CXCL12 to initiate tissue repair by release of IL-5. (3) Atheroprotective functions of ILC2s in PVAT and atherosclerotic plaques are mediated by yet unidentified chemokine-chemokine receptor interactions. (4) ILC2s maintain an anti-inflammatory environment by the release of type-2 cytokines (IL-4, IL-5, IL-13), which leads to recruitment of alternatively activated M2 macrophages and eosinophils in adipose tissues. (5) Increase of pro-inflammatory cytokines (IL-1β, TNFα, IL-6) in adipose tissue inflammation leads to dysfunction of ILC2-mediated responses, which might be mediated by yet unknown chemokine-chemokine receptor signaling. (6) IL-25 treatment leads to expansion of ILC2 exerting atheroprotective functions by the production of B1-secreting IgM cells in atherosclerosis. Innate lymphoid cells type 2 (ILC2), GATA binding protein 3 (GATA3), RAR related orphan receptor A (RORα), myocardial infarction (MI), C-X-C chemokine receptor (CXCR), chemokine (C-X-C motif) ligand (CXCL), CC chemokine receptor (CCR), immunoglobulin M (IgM), Interleukin (IL), Tumor necrosis factor α (TNFα), perivascular adipose tissue (PVAT). Made with biorender.com. The "?" indicates that there is a yet unknown mechanism.

The artery vessels are further composed of media and adventitia, which are imbedded into perivascular adipose tissue (PVAT). It has become clear, that the PVAT plays a major role in the pathogenesis of atherosclerosis. Under homeostatic conditions, PVAT regulates vascular tone and intravascular thermoregulation. However, chronic inflammation (as in atherosclerosis) leads to PVAT dysfunction (36, 37). Of interest, ILC2s have been shown to regulate PVAT homeostasis by secreting type-2 cytokines (IL-4, IL-5, IL-13) in order to maintain an anti-inflammatory milieu by induction of M2 macrophages and eosinophils (**Figure 1**).

LDL receptor deficient (*Ldlr^−/−^*) mouse models are widely used as a model to study an altered cholesterol metabolism due to lack of endocytosis of circulating LDL caused by the LDL-R deletion. Consequently, circulating cholesterol levels are increased, which can be further enhanced when the mice are subjected to a high-fat diet (38). In this regard, first studies in 2015 using immunodeficient *Ldlr^−/−^rag1^−/−^* mouse models showed a protective role of ILC2s in atherosclerosis (39). Specifically, such mice treated with the IL-2/anti-IL-2 complex demonstrated expansion of CD25^+^ ILC2s and a reduction in very low-density-lipoprotein (VLDL) levels. Moreover, an increase in eosinophils in VAT and liver upon ILC2 expansion was observed (39). However, the use of an immunodeficient mouse model and the expansion of ILC2s by IL-2/anti-IL2 complexes rather created an artificial environment.

Therefore, to evaluate the role of naturally occurring ILC2s in atherosclerosis, another group made use of an apolipoprotein e-deficient (*Apoe^-/-^*) mouse model that is susceptible for atherosclerosis development (40). Intriguingly, ILC2s present in PVAT and lymph nodes phenotypically differed from those found in AT. Upon high-fat diet, ILC2s were not only reduced in numbers in PVAT but those remaining also altered their protective phenotype (40). PVAT-resident ILC2s resembled the inflammatory Killer cell Lectin-like receptor G1 (KLRG1)^hi^ ST2^-^ ILC2 (iILC2-like) phenotype and produced less IL-5 and IL-13 (41).

Collectively, these data suggest that ILC2s exerted their anti-inflammatory functions via the production of IL-5 and IL-13, which could be potentially a prerequisite for changes in macrophages and B1-dependent natural IgM production. Importantly, IgM antibodies produced by B1 cells exert atheroprotective properties by capturing lipids expressing oxidation-specific epitopes (OSEs) present on LDL, thereby hindering the uptake by macrophages (42–44).

A strong atheroprotective role of the ILC2-released cytokine IL-5 has been further demonstrated by showing an association of low levels of IL-5 with the presence of plaque development in the carotid bulb in humans (45). To confirm the previous finding in an experimental mouse model, the authors used Western diet-fed *Apoe^-/-^* mice lacking IL-5 and implanted a perivascular shear stress modifier on the right carotid artery to disrupt the pattern of hemodynamic flow. Interestingly, mice that lacked IL-5 displayed enlarged plaques at the location of the shear stress modifier implant, further supporting the importance of IL-5 production by local mediators in order to mediate atherprotective functions (45). The finding that IL-5 has strong atheroprotective functions and that it is mostly produced by ILC2s has been further supported by another study showing that ILC2 depletion in *Ldlr^−/−^* mice led to an acceleration in atherosclerosis progression. Conversely, only wild-type ILC2s could rescue this phenotype in these mice, whereas *Il5^−/−^* ILC2s did not (40).

Myocardial infarctions (MI) cause almost half of the cases of CVDs (46) and after acute MI, an increase of inflammation is induced in order to initiate cell repair. However, persistent inflammation can lead to adverse remodeling of the injured tissue. Several immune cell types migrate to the site of infarction. In this regard, one study found an increased accumulation of ILC2s at sites of MI, which released IL-5 and attracted eosinophils and dendritic cells (DCs) to induce tissue repair (47) (**Figure 1**). Mice with a diphtheria toxin-induced ILC2-specific deletion showed worsened cardiac dysfunction after MI induction, suggesting an important role of ILC2s in repairing tissue injury post-MI (47).

Similarly, Yu et al. examined the role of ILC2s in restoring postischemic injury by using a mouse model of MI with a genetic deletion of ILC2s (48). In wildtype mice, the authors could find an increased number of ILC2s at the site of infarction which contributed to tissue repair as assessed by histology and echocardiography, while genetic deletion of ILC2s impeded this recovery. Importantly, low-dose IL-2 treatment in post-MI mice activated ILC2s and thus, improved the recovery rate of these mice. Of note, this therapeutic approach showed also promising results in a clinical trial of patients with acute coronary syndromes by increased production of IL-5 by ILC2s (48).

As outlined in the previous section, depending on the disease and tissue context, ILC2s express different markers. One study analyzed the role of IL-25-expanded ILC2s isolated from spleens of IL-25-treated *Apoe^-/-^* mice in an *in vitro* and *in vivo* model (49). High-fat diet-fed *Apoe^-/-^* mice were injected subcutaneously with 0.5 × 10^6^ *in vitro*-expanded splenic ILC2s for a total of four transfers with two weeks breaks in between. With this experimental approach, the authors were able to show that the transfer of ILC2s led to a reduction of the lipid content in atherosclerotic lesions of *Apoe^-/-^* mice (**Figure 1**). Interestingly, an increased production of B1-dependent IgM antibodies was observed (49). These findings further highlight the importance of ILC2 interactions with other immune cells ranging from innate to adaptive immunity as well as humoral immunity in order to mediate atheroprotective responses in cardiovascular inflammation.

More recently, it has been proposed that atherosclerosis also has autoimmune-like features. In this regard, it was shown that Tregs and ILC2s directly interact with each other during cardiovascular inflammation (50). Specifically, the disruption of cell-to-cell contacts reduced the release of IL-13 by ILC2s *in vitro* and *in vivo*. The importance of IL-13 in protecting against cardiovascular inflammation has been highlighted in previous sections.

Intriguingly, ILC2 (18, 19) and Treg numbers [summarized in (51)] are reduced in atherosclerotic mouse models and humans with CVDs. Moreover, Tregs from *Apoe^-/-^* mice have been shown to have a reduced ability to suppress CD4^+^ T cell proliferation *in vitro* (52). Notably, under conditions of sustained inflammation such as in atherosclerosis, Tregs have been shown to acquire a pro-inflammatory phenotype (53). A similar ILC2 plasticity has also been observed in airway diseases (54–56). However, the exact mechanisms leading to reduced numbers of ILC2s in atherosclerosis remain unclear. Moreover, understanding mechanisms involved in the Treg-ILC2 are still in its infancy.

Most of the studies used the *Rora^fl/fl^Il7r^Cre/+^* mouse model for the generation of ILC2 deletion, however, other ILC subsets might be affected in this model as well (57). Therefore, new mouse lines that specifically target or delete ILC2s are currently being investigated in order to improve the specificity (58–60).

Overall, these studies could demonstrate that ILC2s play an important role in protecting against vascular inflammation. However, under conditions of sustained inflammation, ILC2s acquire an inflammatory phenotype in atherosclerosis. Therefore, a better understanding of underlying mechanisms that shape ILC2 responses under such conditions are needed.

## ILC2s and chemokine receptors/ligands in disease

Chemokines and chemokine receptors play an important role in recruiting immune cells to sites of inflammation and therefore significantly impact on disease progression. Importantly, while chemokine receptors can have multiple ligands and vice-versa chemokines can bind to more than one receptor, they only pair with the structurally identical cysteine residues (3). However, depending on the pairing, chemokines and their receptors can exert different effects. In the past years, it has become clear that chemokine-chemokine receptor signaling is not only essential for leukocyte recruitment in atherosclerosis, but that their signaling cascade also mediates other important biological functions. For instance, blockade of chemokine (C-C motif) ligand 19 (CCL19) and CCL21 binding to CC chemokine receptor 7 (CCR7) has been shown to preserve foam cell content (61). Moreover, depending on the cells they are expressed on, chemokine-chemokine receptor interactions contribute to their pleiotropic function of either mediating pro- or anti-inflammatory responses (62).

Some early studies in the 2000’s already describe how chemokine receptors are able to shape T cell subsets and their responses. During that time, the existence of ILCs had not been known yet. One could therefore speculate, that those studies describing “uncommitted, primed, precursor cells” (Thpp), misidentified first interactions of ILC2s as being Th2 precursors with chemokines (63, 64).

**Figure 2:**
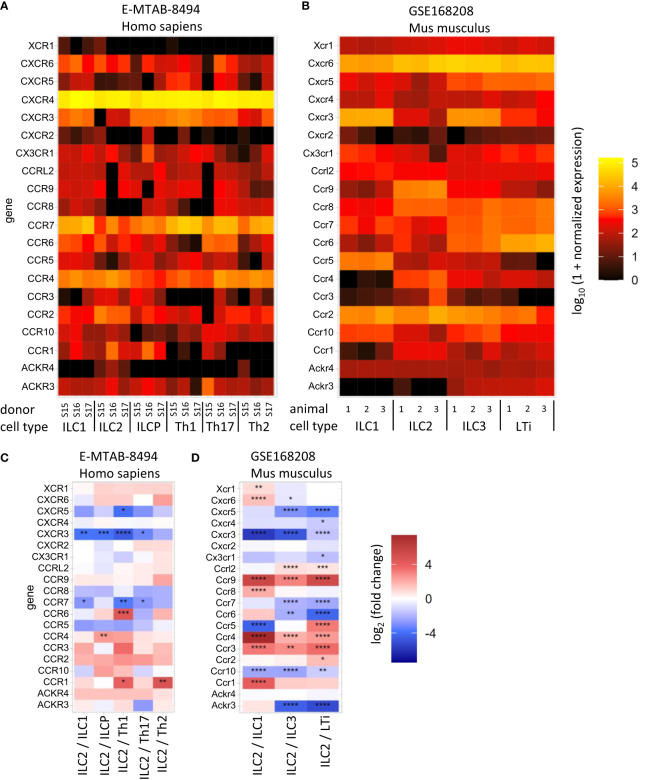
Heat maps show normalized mRNA copy number **(A, B)** or the expression difference in the indicated cell types **(C, D)** of the selected chemokine receptor-coding genes. The data from human **(A, C)** and mouse **(B, D)** cells are available at ArrayExpress E-MTAB-8494 (72) and GEO GSE168208 (73), respectively. Innate lymphoid cell (ILC), helper T cell (Th), Lymphoid tissue inducer (LTi), ILC precursor (ILCP). The analysis was performed as described in (73). P-value notation: * 0.01 ≤ p < 0.05, ** 0.001 ≤ p < 0.01, *** 0.0001 ≤ p < 0.001, ****p < 0.0001.

Currently, several chemokine receptors expressed on ILC2s have been identified: CCR9, C-X-C chemokine receptor 4 (CXCR4), CXCR6 (15, 65–67), CCR4 (68) and CCR8 (69) in mice, and the expression of some chemokine receptors (CCR2, CCR4, CCR10 CXCR4) on ILC2s have also been described in humans, although studies remain scarce (70, 71) (**Figures 1**, **2**).

With respect to ILC2s, for instance, in response to allergens, IL-13 release by ILC2s leads to the production of DC-derived CCL17, thereby stimulating Th2 cell responses (26). Conversely, binding of CCL1 to CCR8 expressed on ILC2s protected mice from intestinal damage in a model of experimental colitis (74). Moreover, it has been shown that CCL1-CCR8 signaling on ILC2s triggered their proliferation and increased their ability to respond to helminth infections (68). In humans, CCR10^+^ ILC2s have been shown to expand upon severe acute respiratory syndrome coronavirus 2 (SARS-CoV-2) pneumonia to trigger recovery and tissue repair and their increased frequency was negatively correlated with pro-inflammatory markers (75).

With respect to vascular inflammation, CXCR4 expressed on vascular cells as well as on B cells exerts protective functions in atherosclerosis (44, 76). In this regard, it has been shown that ILC2s are recruited via CXCR4-CXCL12 interaction and aid in the recovery from experimental MI in mice by increasing the levels of IL-5 (48) (**Figure 1**).

Further, CCR9-CCL25 signaling has been shown to be pro-atherogenic, since inhibition of CCR9 led to a delay in the development of atherosclerosis in *Apoe^-/-^* mice (77). However, the exact functions of CCR9 expressed on ILC2s in the context of ACVDs remain elusive. So far, it has only been reported that CCR9 might serve as trafficking receptor for ILC2s to several organs (65). Similarly, CXCR6 is an important T-cell homing receptor to the aortic wall where it accelerates atherosclerosis (78) but its role on ILC2s in ACVDs still needs to be explored. So far, it has only been shown that CXCR6 is crucial for ILC progenitors to egress from the bone marrow at steady state conditions (79).

Similarly, disrupted CCL1-CCR8 signaling in Tregs has been shown to exacerbate atherosclerosis in mice (80). Specifically, a reduction of the anti-inflammatory cytokine IL-10, and reduced Tregs could be observed in aorta and spleen of fat-fed *Apoe^-/-^* mice deficient in CCL1. Importantly, this effect could also be mimicked by blocking CCR8 in high-fat-fed *ldlr^−/−^* mice, as evidenced by decreased recruitment of Tregs to the aorta (80).

Importantly, CCR8 is also highly expressed on ILC2s (69). However, studies investigating the role of CCR8 on ILC2s in atherosclerosis are still lacking.

A list of known shared chemokine receptors between ILC2s and other immune cell subsets is shown in **Table 1**.

**Table 1:** ILC2-immune cell crosstalk and their function in atherosclerosis.

Chemokine receptor	shared expression with ILC2s	Ligands	Role in atherosclerosis	References
CCR4	Th2 cells, Tregs	CCL17 CCL22	Atheroprotective *Recruitment of Tregs to lesions*	(68, 81)
CCR8	Tregs	CCL1	Pro-atherogenic *Suppression of Tregs*	(80, 82)
CCR9	DCs, B cells, T cells	CCL25	Pro-atherogenic *Increased recruitment of pro-inflammatory immune cells*	(77)
CXCR4	B cells, NK cells	CXCL12	Atheroprotective *Recruitment of immune cells important for tissue repair and increased plasma IgM levels produced by B cells*	(44, 48, 76)
	Endothelial cells		Pro-atherogenic *Lesion development*	
CXCR6	Th17 cells	CXCL16	Pro-atherogenic *Recruitment of pro-inflammatory IL-17A-producing T cells into aortas*	(78, 83)

## Future perspectives

Due to their local availability, ILC2s are an attractive target for the development of novel therapies. Accordingly, some strategies to therapeutically manipulate ILC2s in disease have already been developed or are currently being investigated in clinical trials [see (84, 85)]. For instance, for the treatment of asthma there are several FDA-approved drugs like mepolizumab, benralizumab, and reslizumab that target the cytokine IL-5, which is expressed by ILC2s, among others (86). Current therapies using monoclonal antibodies targeting IL-5 are in clinical use as add-on therapy for the treatment of uncontrolled asthma (87). In this setting, IL-5 blockade is required to dampen hyperactivity in asthma by reducing recruitment of eosinophils and therefore, it is not specific to a given cell type but it is applied systemically. For the treatment of specific diseases where the role of ILC2s is implicated (e.g. CVDs), future studies are required that aim at specifically targeting ILC2-mediated IL-5 secretion.

In CVD, IL-2 treatment has led to promising results in mice by the expansion of ILC2s and hence, promoting their atheroprotective function (39) and recovery rate in post-MI mice (48). Since data from murine studies point towards an atheroprotective role of ILC2s and Tregs, using low-dose IL-2 treatment with the goal to expand both immune cell subsets could be a promising approach to dampen inflammatory responses in early atherosclerosis. The current literature focused on the effects of IL-2/anti-IL-2 treatment in expanding either Tregs (88, 89) or ILC2s (40, 90) in a specific disease setting. These studies did not investigate the expansion of both cell types or others that might be expanded upon this treatment strategy. It would be therefore crucial to understand which cell types become activated upon that treatment and how IL-2-mediated expansion affects their phenotypes.

In this regard, the phase 2 clinical trial called IVORY has been started to investigate the beneficial effect of low-dose IL-2 therapy in patients with acute coronary syndromes (ACS), which will be completed in 2024 (91).

Interestingly, whereas treatment of high fat-fed *Apoe^-/-^* mice with IL-33 reduced atherosclerosis development (92), a more recent study showed that ILC2s can also mediate the IL-33-induced eosinophilic pericarditis (93). Here, anti-IL-5 treatment led to a reduction of infiltrating eosinophils into the heart during pericarditis (93).

These studies suggest that therapeutical targeting of the IL-33/ST2/ILC2s signaling axis could be used as potential treatment for several CVDs and highlight the importance to investigate the function of ILC2-mediated signaling pathways in a disease-dependent context.

Notably, systemically administered drugs have general safety concerns due to off-target effects. Thus, targeting tissue-resident ILC2s and/or their signaling cascade via chemokine-chemokine receptor interactions enables a more specific and efficient approach for therapeutic manipulation. Hence, a deep phenotypic characterization of ILC2 is required for a better understanding of their mode of action in a disease context such as ACVDs.

Along these lines new technologies such as single cell RNA (scRNA) or nuclei RNA (snRNA) sequencing could improve our understanding of the state of ILC2 in a disease- and tissue-specific manner. ScRNA sequencing has identified ILC2 cells among human lymphocytes (94), and characterized them based on their microenvironmental localization in steady state and disease in several human tissues (95, 96). Importantly, Jiang et al. identified new ILC2 subclusters in myocardial ischemia of mice (97) and another study using scRNA sequencing revealed heterogeneity in the immune phenotypes of ILC2s from lesional atopic dermatitis skin in humans (98). These studies highlight the potential of scRNA sequencing technologies to advance our understanding in the heterogeneity of ILC2 subset phenotypes in healthy and diseased conditions. Moreover, the identification of such subsets will critically contribute to the development of therapeutic applications.

## Author contributions

MK: Writing – original draft. EV: Writing – review & editing. AS: Writing – review & editing. CW: Writing – review & editing. YD: Writing – review & editing.
